# Supplementary material for: Addressing a Pre-Clinical Pipeline Gap: Development of the Pediatric Acute Myeloid Leukemia Patient-Derived Xenograft Program at Texas Children’s Hospital at Baylor College of Medicine
Source: Biomedicines. 2024 Feb 8;12(2):394. doi: 10.3390/biomedicines12020394 (PMC10886789; doi:10.3390/biomedicines12020394)
Supplement: Supplementary file 1 [file biomedicines-12-00394-s001.zip › biomedicines-2779056-supplementary.pdf]

A – AML901

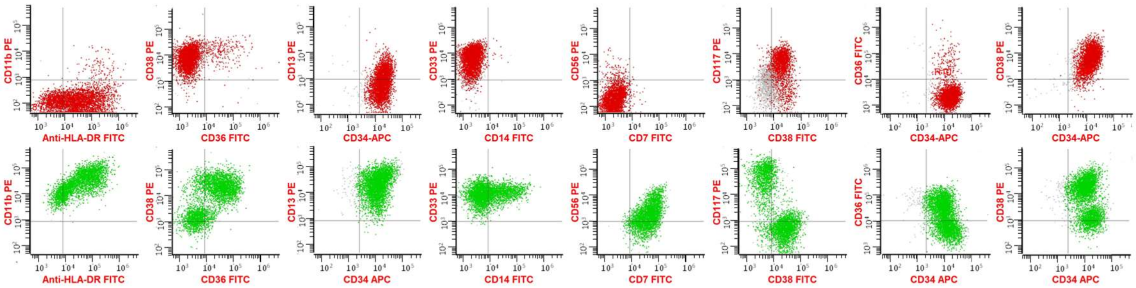

B – AML902

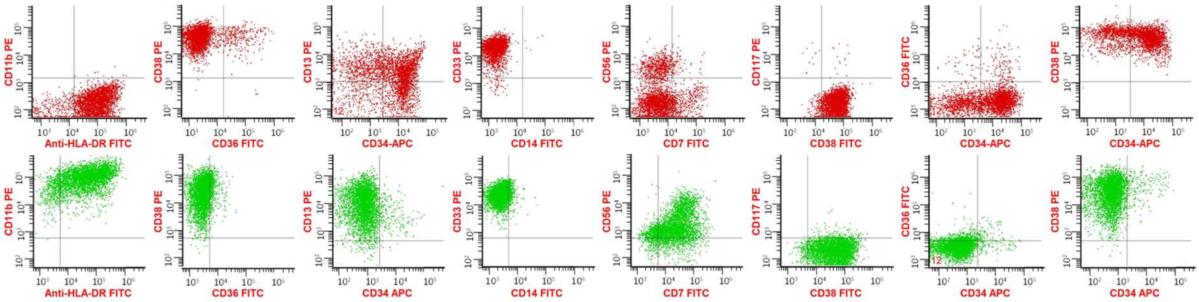

C – AML903

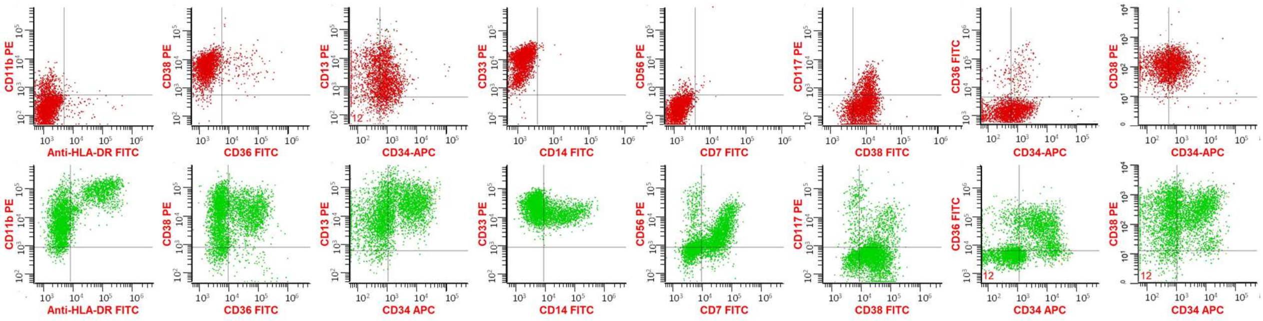

D – AML904

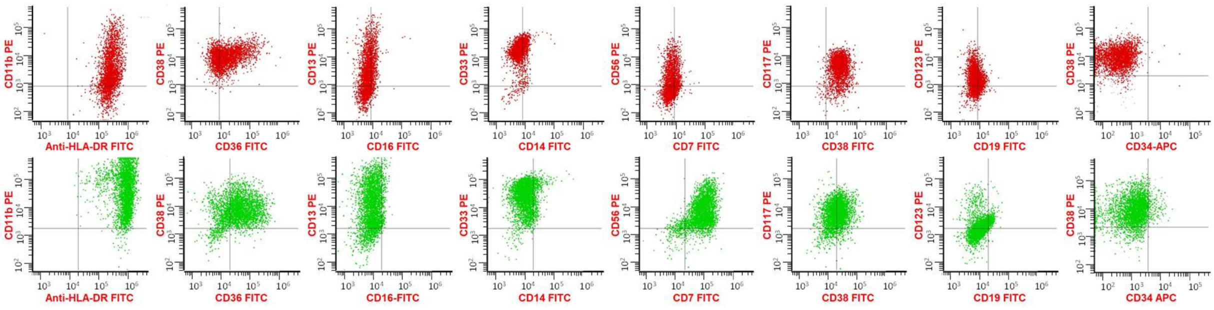

E – AML905

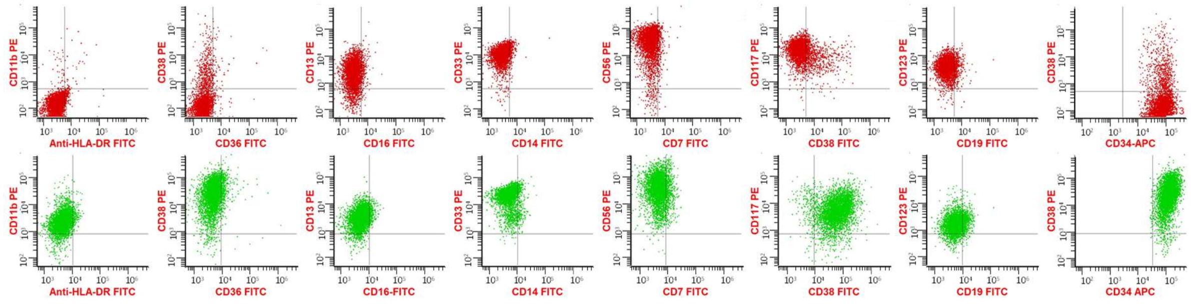

F – AML906

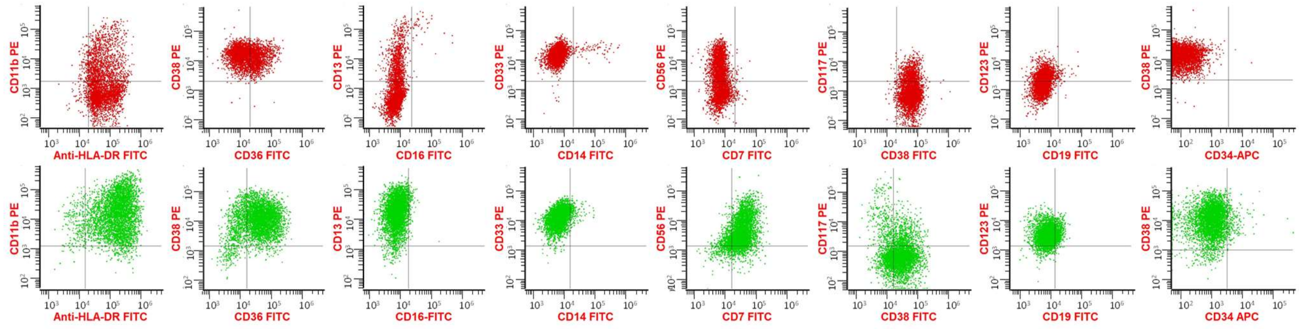

Supplementary Figure S1A-F. Top row (red) depicts cell surface antigenic expression in a diagnostic specimen. Bottom row (green) depicts the PDX cell line derived from the same diagnostic cells. Axes are in number of cell surface antigenic molecules. AML901 (A), AML902 (B), AML903 (C), AML904 (D), AML905 (E), AML906 (F).

Supplementary Table S1A. List of genes targeted for mutations and copy number analysis

ABL1	ABL2	AKT1	AKT2	ALK	ANK3	APC	ARAF
ARID1A	ASXL1	ASXL2	ATM	ATRX	BAZ1A	BCL11B	BCOR
BCORL1	BIRC6	BRAF	CALR	CBL	CBLB	CCND1	CCND2
CCND3	CCT6B	CDKN1B	CDKN2A	CDKN2B	CEBPA	CHD4	CNOT3
CREBBP	CRLF2	CSF1R	CSF3R	CTCF	CUX1	DDX3X	DDX41
DHX15	DNM2	DNMT3A	ECT2L	EED	EIF6	ELF1	EP300
EPOR	ERG	ETNK1	ETV6	EZH2	FAT1	FAT4	FBXW7
FLT3	FPGS	GATA1	GATA2	GATA3	GNA13	GNAS	H3F3A
HDAC9	HRAS	HUWE1	ID3	IDH1	IDH2	IKZF1	IKZF2
IKZF3	IL7R	JAK1	JAK2	JAK3	KANSL1	KAT6B	KDM5A
KDM6A	KIT	KMT2A	KMT2C	KMT2D	KRAS	LEF1	MAP2K1
MBNL1	MED12	MGA	MLH1	MLLT3	MPL	MSH2	MSH6
MYB	MYC	MYCN	MYD88	NF1	NIPBL	NOTCH1	NOTCH2

NOTCH3	NPM1	NR3C1	NR3C2	NRAS	NSD2	NT5C2	ORAI1
PAX5	PCBP1	PDGFRA	PDGFRB	PHF6	PHIP	PIK3C2A	PIK3CA
PIK3CD	PIK3R1	PMS2	PPM1D	PRDM2	PRPS1	PRPS2	PTEN
PTPN11	PTPRC	RAD21	RB1	RELN	RHOA	RIT1	RPL10
RPL5	RUNX1	SAMD9	SAMD9L	SETBP1	SETD2	SETX	SF3A1
SF3B1	SH2B3	SMARCA4	SMARCB1	SMC1A	SMC3	SOS1	SRSF2
STAG2	STAT3	STAT5B	SUZ12	SYNE1	TERT	TET1	TET2
TNFRSF14	TP53	TSPYL2	U2AF1	U2AF2	UBTF	USP7	USP9X
VPREB1	WT1	ZBTB7A	ZEB2	ZFHX3	ZRSR2		

Supplementary Table S1B. List of genes targeted for detecting gene fusions.

ABL1	ABL2	AFDN	ALK	BCL11B	BCL2	BCL6
BCR	BIRC3	BLNK	BRAF	CBFA2T3	CBFB	CCND1
CDK6	CHD1	CHIC2	CIITA	CREBBP	CRLF2	CSF1R
CTLA4	DEK	DGKH	DUSP22	EBF1	EIF4A1	EPOR
ERG	ETV6	FEV	FGFR1	FGFR3	FLT3	FOXP1
FRK	FUS	GLIS2	HOXA10	IKZF1	IKZF2	IKZF3
IL2RB	IL3	ITK	JAK2	KAT6A	KIT	KLF2
KMT2A	LYN	MALT1	MECOM	MEF2D	MKL1	MLF1
MLLT1	MLLT10	MLLT3	MN1	MYB	MYC	MYH11
NF1	NFKB2	NOTCH1	NTRK1	NTRK2	NTRK3	NUP214
NUP98	PAG1	PAX5	PBX1	PDCD1LG2	PDGFRA	PDGFRB
PICALM	PML	PRDM16	PTK2B	RARA	RARB	RBM15
RET	ROS1	RUNX1	RUNX1T1	SEMA6A	SETD2	SPI1
STIL	SYK	TAL1	TAL2	TBL1XR1	TCF3	TCL1A
TFG	TP63	TP63	TSLP	TYK2	ZCCHC7	ZNF384
